# Supplementary material for: Orexin effect on physiological pulsations of the human brain
Source: Proc Natl Acad Sci U S A. 2025 Aug 1;122(31):e2501578122. doi: 10.1073/pnas.2501578122 (PMC12337265; doi:10.1073/pnas.2501578122)
Supplement: Supplementary file 1 — Appendix 01 (PDF) [file pnas.2501578122.sapp.pdf]

PNAS

Supporting Information for

Orexin Effect on Physiological Pulsations of the Human Brain

Matti Järvelä[1,2,3], Janne Kananen[1,2,3,4], Heta Helakari[1,2,3], Vesa Korhonen[1,2,3], Niko Huotari[1,2,3], Tommi Väyrynen[1,2,3], Katariina Hautamäki[1,2,3], Lauri Raitamaa[1,2,3], Johanna Tuunanen[1,2,3], Mika Kallio[2,3,4], Johanna Piispala[2,3,4], Hanna Ansakorpi[5,6], Vesa Kiviniemi[1,2,3]*

1 Oulu Functional Neuroimaging, Department of Diagnostic Radiology, Oulu University Hospital, Oulu, Finland

2 Medical Research Center (MRC), Oulu University Hospital, Oulu, Finland

3 Research unit of Health Sciences and Technology, the Faculty of Medicine, University of Oulu, Oulu, Finland

4 Clinical Neurophysiology, Oulu University Hospital, 90220 Oulu, Finland

5 Research Unit of Neuroscience, Neurology, University of Oulu, Oulu, Finland

6 Department of Neurology, Oulu University Hospital, Oulu, Finland

Corresponding author: *Vesa Kiviniemi, Prof, MD

Email: vesa.kiviniemi@oulu.fi

This PDF file includes:

Supporting text 1 to 3
Figures S1 to S20
Tables S1 to S7

Other supporting materials for this manuscript include the following:

Dataset S1 and S2 (separate files)

Supporting Information Text 1

Pulsatile water flow induces rapid and prominent amplitude oscillations in the MREG time signal

Pulsatile flow of CSF should induce signal oscillations in a T2*-weighted MRI sequence like MREG as movement of water protons induces loss of spin phase coherence (1, 2). We find in the phantom study that increasing flow speeds induced by faster water pumping frequency are detectable from the MREG time signal amplitude oscillations, power spectrum, and spectrogram (Fig. 3A). Time signal oscillations gain amplitude and become sharper when flow speed is increased. This is reflected in the power spectrum, where the main flow frequency dominates, with increasing power corresponding to higher flow speeds (from 1.9 Hz to 4.4 Hz, while the 1.0 Hz peak is a signal artefact from the MRI-scanner helium pump). Of note, when the mean flow speed is kept stable, the signal amplitude oscillations and spectral power accumulation also remain stable as reflected by the narrow principal peaks occurring while flow is turned on (Fig. 3A periodogram). The mean flow of 7 cm/s induced the first decree harmonic seen in the spectrogram (from 80 s to 140 s), while the two higher flow speeds (mean 14 cm/s from 140 s to 200 s, and 21 cm/s from 200 s onwards) present heterodynes produced by the low frequency envelope oscillation that was induced by the high maximal flow of water inside the system. Note that the higher frequency heterodyne of the 4.4 Hz principal frequency is aliased, as it would locate around 5.6 Hz, thus exceeding the critical sampling rate of the MREG. The same increase as a function of increasing flow speed is captured in the spectral domain by SP and in the time domain by CV (Fig. 3B). In SP and CV, the increases from baseline (no flow) to 7 cm/s flow were the most prominent, with lower incremental increases from the lower to higher flow speeds (Fig. 3B, Table 1). SE first decreased sharply from baseline to 7 cm/s mean flow, yet remained stable from 7cm/s to 14 cm/s, while increasing from 14 cm/s to 21 cm/s flow rate (Fig. 3B, Table 1). We suppose that the stable SE between 7cm/s to 14 cm/s is due to the model producing only one pulsing phenomenon, i.e. inducing power only to one narrow frequency range at a time, while the increase in SE between 14 cm/s to 21 cm/s relates to the high maximal flow speeds which cause rapid signal oscillations that exceed the critical sampling rate threshold of MREG (5 Hz), thus leading to aliasing (3). Furthermore, these higher flow speeds may

cause laminar flow to turn turbulent inside the phantom model, which would include many different flow rates compared to linear flow thus increasing the SP across the whole spectrum. Indeed, visually in the Fig. 3A spectrogram, most power across the full 5 Hz frequency range seems to be present in the 21 cm/s flow time-frame, which is confirmed by our result that the median background SP at 21 cm/s flow is 12.8 times greater compared to baseline, and 1.6 times greater compared to the 14 cm/s flow. This suggests that even faster sampling would be beneficial when operating with high flow systems. We conclude that the MREG-derived biometrics calculated here are fit to capture pulsatile flow-related T2* effects, where CV measures time signal oscillations, SP indicates spectral domain power, and SE shows how the spectral content is distributed across the system.

Supporting Information Text 2

Sensitivity analysis of within awake HC brain pulsations

As a sensitivity analysis, we tested whether the brain pulsations differed within the awake HC group, as this could affect our further analyses. Here, we split the awake HC group into two subgroups matched for age and sex (group 1: n = 40, mean age 37.6 ± 16.2, 26 females; group 2: mean age 37.1 ± 15.5, 26 females), and compared their CV/SP/SE brain maps. These were then compared with FSL randomise (4) using 10 000 iterations and correcting for age and sex in the design.

Sensitivity analysis of medication effects and arousal:

We first tested for the differences between the medicated and nonmedicated NT1 patients in mean cardiac frequency, MAP, systolic-, and diastolic pressures as well as mean absolute and relative motion parameters, as differences in these may influence brain pulsation analyses. We found no significant differences (Table S3).

To determine whether the NT1 group medication status influences our results, we first identified any significant comparison where NT1 group was present (Figures S2, S4, S6, S7, S9, S11, S13, S15, S16 so in nine cases). From these maps, we extracted the mean pulsation biometrics

(CV/SP/SE, one value per patient) in all pulsation frequencies (VLF and cardiorespiratory frequencies) with FSL tools. The NT1 patients with 0 to 1 medications (n = 9, mean age 27.9 ± 10.4 years, 3 females) and 2 to 3 medications (n = 12, mean age 28.3 ± 8.62 years, 9 females) were grouped and tested for differences (Table S7, Fig. S18). We found no significant differences suggesting that medication does not induce increased or decreased mean level of brain pulsation in our data set.

To further explore if medication affects our results, we explored if the voxel-wise pulsations differ between the medicated and nonmedicated NT1 patients (nonmedicated n = 3, mean age 31.3 ± 3.79 years, 2 females and medicated n = 18, mean age 27.6 ± 9.74 years, 10 females). We extracted and compared the voxel-wise brain pulsation biometrics in all pulsation frequencies from the same significant pulsation maps as above. Furthermore, to investigate whether medication has a positive or negative effect (i.e. increasing or decreasing) on the brain pulsations in the NT1 group, we created an overall medication effects model (binarized medication status where no medications corresponds to 0 and any medication corresponds to 1), and a model weighted by the number of medications [i.e. weighting of the estimates as a function of medical burden: nonmedicated (n = 3), patients with 1 medication (n = 6), patients with 2 medications (n = 10) and patients with 3 medications (n = 2)]. To infer significant voxels, we used FSL randomise (4) using full number of iterations and implementing family-wise error-corrected threshold-free cluster enhancement correction while correcting for age and sex in the design. We found no voxel-wise differences between the nonmedicated and medicated NT1 patients, and no significant overall medication effect in our dataset. However, in the weighted model, we found significant increase by medication burden in a small area of VLF CV (145 voxels against the original 3572 voxels) and SP (105 voxels against the original 329 voxels, Fig. S17) while cardiorespiratory pulsations remained unaffected. This suggests that in these voxels, medication may increase vasomotor pulsation.

To examine whether medication increases arousal, and thus through arousal affects the same brain pulsation biometrics discussed above, we compared the nonmedicated and medicated NT1

patients while regressing out the effect of medication status. We hypothesized that given the assumption that medicated patients would be more awake than their nonmedicated counterparts, this should be reflected as differences in the pulsation biometrics. The assumption is, that when there are no differences in movement, blood pressure measurements and mean cardiac frequency, and medication status is controlled for (as well as sex and age) in the model, the main component that would drive possible differences between the medicated and nonmedicated NT1 patients' brain pulsation is arousal. This is reasonable, as we have earlier shown that low arousal state in NREM sleep greatly increases all brain pulsations (5). Thus, this model indirectly measures whether the arousal state between the medicated and nonmedicated NT1 patients differs. To infer significant voxels, we used FSL randomise as described above but also correcting medication in the design. We found no significant voxels suggesting that in our dataset, the nonmedicated and medicated NT1 patients have no significant differences in brain pulsations via arousal state.

To explore the arousal states between awake HC, NT1 group and sleeping HC, we calculated the HRV RMSSD from available photoplethysmogram data (awake HC n = 48, NT1 group n = 14, sleeping HC n = 11, please see methods section in the manuscript). We found no significant differences between the NT1 group and awake HC, but as expected higher RMSSD in sleeping HC compared to both other groups (compared to NT1 $p = 0.0032$, and to awake HC $p = 0.0016$). This suggests that the NT1 patients in our dataset are not at a heightened arousal state nor are they asleep. We also used a general linear model (implemented in R) to explore whether medication status increases or decreases RMSSD in the NT1 group. We found no significant increase or decrease in RMSSD values in the NT1 group as per medication ($p = 0.63$, 95% CI = -2.9 to 1.7, estimate = -0.60, R2 = 0.30). This suggests that medication status does not affect the arousal state in our NT1 population.

Finally, we had EEG data (equal to that used for the HC NREM sleep group) from 9 NT1 patients that were later imaged for the present study and 7 awake HC, which we used to study the ability of a subpopulation of our NT1 to remain awake for the required 5 min imaging time. The international

10-10 system was used to choose channels for further analysis. Bad channels were excluded based on the following criteria: standard deviation exceeding 2000 μV, average correlation with neighboring electrodes falling below 0.1, or electrode impedance surpassing 1 MΩ. Spherical interpolation was used to replace the removed channels. MATLAB was used to calculate spectrograms and alpha (8-12 Hz) and beta (12.5-30 Hz) frequency powers which were compared between the two groups.

The fast beta band (12.5 – 30 Hz) power is known to decrease while the alpha band (8 – 12 Hz) power increases during the eyes-closed condition (6). As our subjects were instructed to remain awake eyes fixating on a cross, the alpha and beta power can give an estimate of the arousal. Thus, if the NT1 patients are unable to remain awake, there should be a difference in these bands' power compared to that of the awake HC. We calculated the mean alpha (NT1 median with interquartile range = 40 [26 - 74], HC median = 36 [30 - 58]) and beta power (NT1 median = 43 [35 - 72], HC median = 33 [30 - 58]) across the 10-10 channels for a period of 5 min. We found no differences in these powers between the NT1 patients and awake HC (alpha power $p = 0.92$, beta power $p = 0.68$), though visually more fluctuation in the powers across the session was present in the NT1 (Fig. S20 spectrogram examples). This suggests indirectly that at least a proportion of our NT1 group can maintain wakeful rest even with fluctuation in the arousal state without falling asleep for the required 5 min period.

Regarding arousal in out NT1 group, we further note that: 1) we verbally checked the vigilance state of the patients at the end of scanning, 2) used the first 5 minutes of the scan, when the patients were most alert, 3) showed that ROC analysis separated the HC NREM sleep and NT1 groups with high accuracy, 4) especially the cardiac pulsations were in the opposite direction to that expected if the NT1 group patients were indeed sleeping, and 5) there were no differences in mean cardiac frequency between the NT1 patients and awake HC, but as would be expected, the HC NREM sleep group had significantly lower heart rate compared to the NT1 and awake HC groups.

As a summary, our analyses on medication effect show that in our dataset of NT1, only on a small area is affected via medication by an increase in VLF CV and SP while other pulsation frequencies remain unaffected (Fig. S17). Even the exclusion of these voxels would not change the interpretation of the original results. Our analyses on the arousal state in the NT1 group consistently suggest that the patients are not in a state of heightened arousal nor are they asleep, and that the medication status does not predict for increasing or decreasing arousal state.

Supporting Information Text 3

Sensitivity analysis statistics

Visual estimation and the Shapiro-Wilk test were used to examine data normality. NT1 patients' mean CV and SP in the VLF and cardiac frequencies and the full band SE were normally distributed ($n = 9$ with 0 to 1 medications and $n = 12$ with 2 to 3 medications. Normality with Shapiro-Wilk: VLF mean CV and SP $p = 0.75$ and $p = 0.12$; cardiac mean CVs $p = 0.40$ and $p = 0.21$ and SP $p = 0.16$; full band mean SEs $p = 0.75$ and $p = 0.80$), and thus compared with a T-test. Respiratory CV and SP (Shapiro-Wilk $p = 0.017$ and $p = 0.00032$) as well as EEG mean alpha and beta powers (Shapiro-Wilk $p = 3.5E-6$ and $p = 1.0E-7$) did not follow normal distribution, so Wilcoxon Rank-Sum Test was used in these comparisons. For differences across the three groups in HRV, awake HC ($n = 38$), NT1 ($n = 14$), and HC NREM sleep ($n = 11$) HRVs were found normally distributed (Shapiro-Wilk awake HC $p = 0.12$, NT1 $p = 0.88$, HC NREM sleep $= 0.29$) and compared with one-way ANOVA followed by the pairwise Tukey's Honest Significant Difference test. A general linear model was used to model the medication effect on HRV RMSSD in the NT1 group, where nonmedicated ($n = 2$), the group with 1 medication ($n = 4$), and the group with 2 medications ($n = 8$). As the NT1 group MAP, systolic-, diastolic pressure and mean cardiac frequency were normally distributed (Shapiro-Wilk $p = 0.62$, $p = 0.55$, $p = 0.17$, $p = 0.34$ respectively), a T-test was used to compare these values between nonmedicated ($n = 3$) and medicated patients ($n = 17$ for blood pressure measurements and $n = 18$ for mean cardiac frequencies). Mean absolute and relative motion in the NT1 group was nonnormally distributed (Shapiro-Wilk $p = 0.019$ and $p = 0.0073$), so

Wilcoxon Rank-Sum test was used to compare nonmedicated (n = 3) and medicated patients (n = 18). FSL's randomise, which uses conditional Monte Carlo random permutations implementing family-wise error-corrected threshold-free cluster enhancement correction (4) that results in multiple comparisons corrected p-value maps, was used to 1) compare all brain pulsation (CV/SP/SE in VLF and cardiorespiratory frequencies) in nonmedicated (n = 3) and medicated NT1 patients (n = 18), 2) to model the overall positive or negative effect of medication to brain pulsation (CV/SP/SE in VLF and cardiorespiratory frequencies) in the NT1 group [(nonmedicated (n =3) and medicated (n = 18)], 3) to model the weighted effect of medication to these same brain pulsations [i.e. nonmedicated (n = 3), patients with 1 medication (n = 6), patients with 2 medications (n = 10), patients with 3 medications (n = 2)], and 4) to compare these same brain pulsations between the nonmedicated (n = 3) and medicated patients (n = 18) in a model where assumed medication effect is regressed out. All randomise models controlled for age and sex. Significant threshold for all tests: $p < 0.05$.

Supporting Figures and Tables

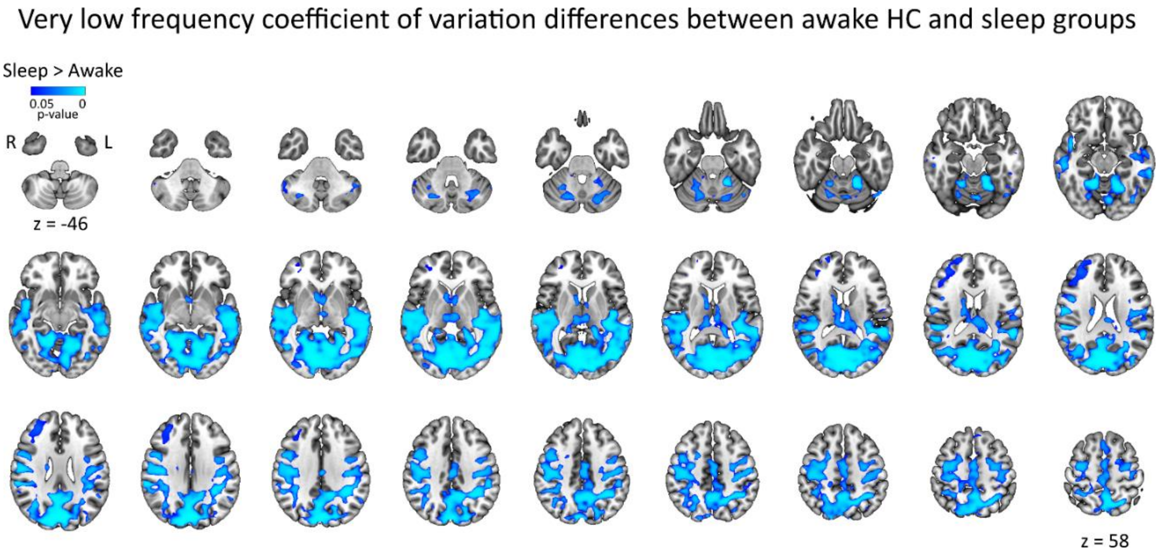

Fig. S1. Spatial distribution of the very low frequency coefficient of variation difference maps between the awake HC and NREM sleep groups (NREM sleep > awake HC). R = right, L = left, z = axial plane MNI152 coordinate.

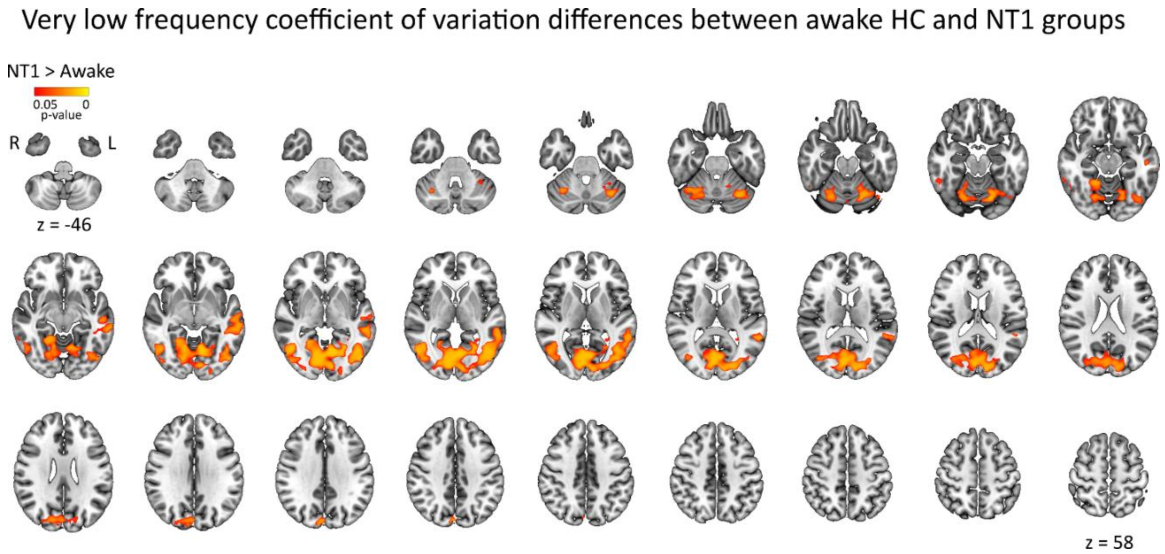

Fig. S2. Spatial distribution of the very low frequency coefficient of variation difference maps between the awake HC and NT1 groups (NT1 > awake HC). R = right, L = left, z = axial plane MNI152 coordinate.

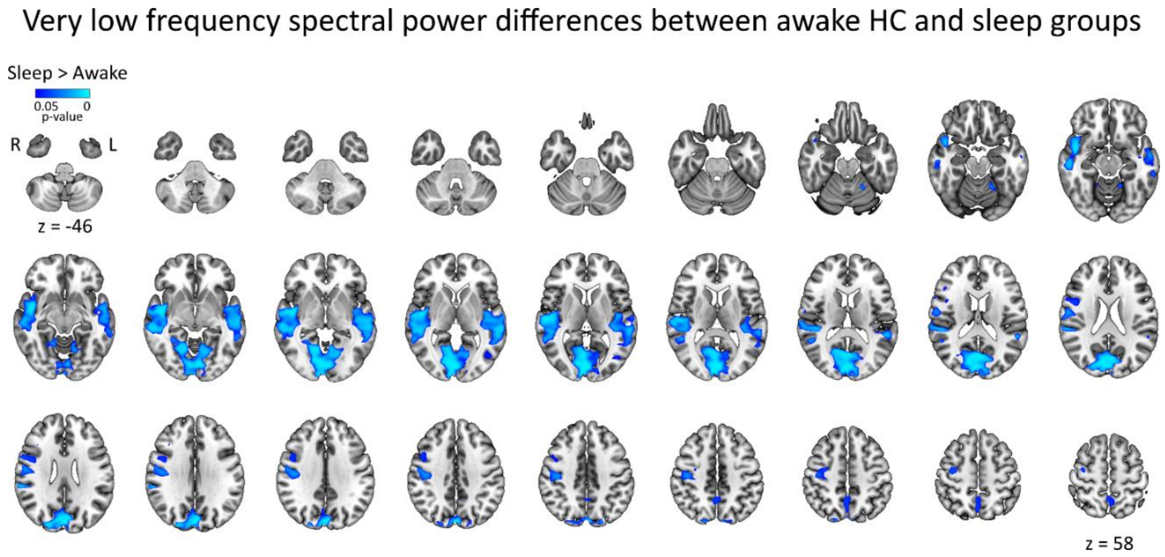

Fig. S3. Spatial distribution of the very low frequency spectral power difference maps between the awake HC and NREM sleep groups (NREM sleep > awake HC). R = right, L = left, z = axial plane MNI152 coordinate.

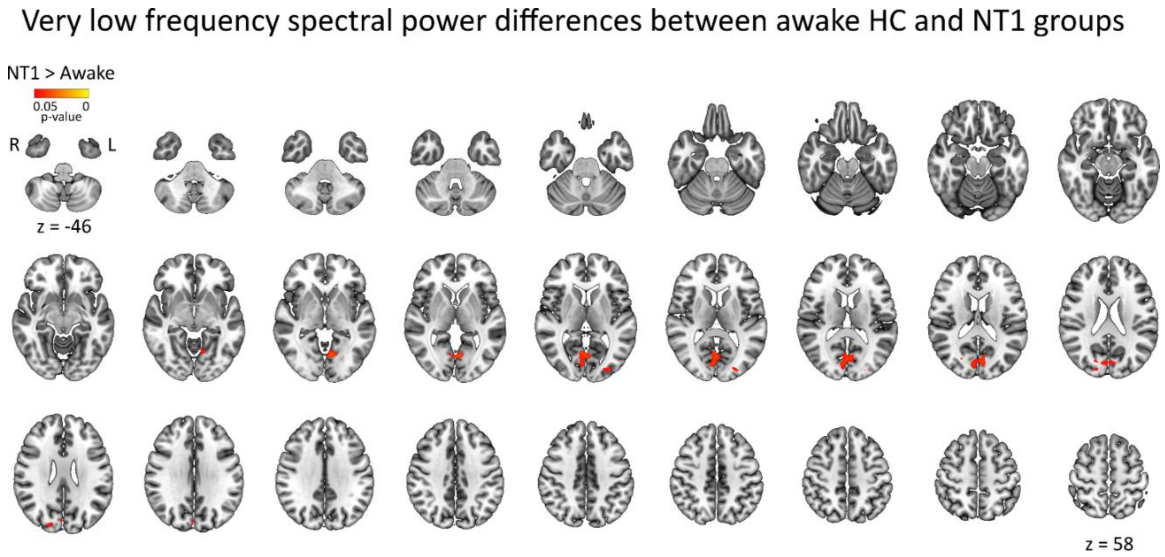

Fig. S4. Spatial distribution of the very low frequency spectral power difference maps between the awake HC and NT1 groups (NT1 > awake HC). R = right, L = left, z = axial plane MNI152 coordinate.

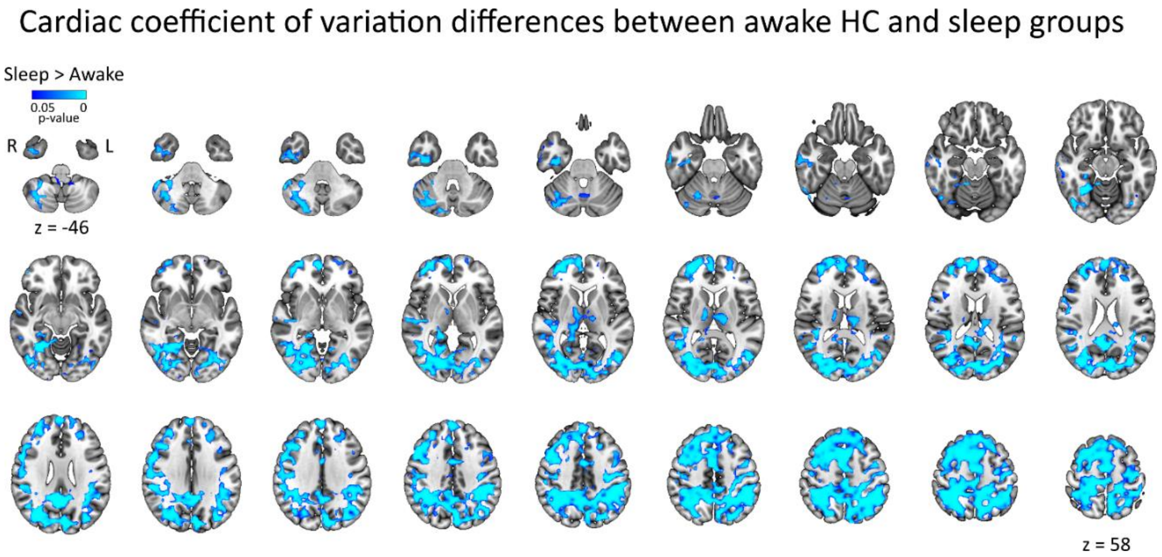

Fig. S5. Spatial distribution of the cardiac coefficient of variation difference maps between the awake HC and NREM sleep groups (NREM sleep > awake HC). R = right, L = left, z = axial plane MNI152 coordinate.

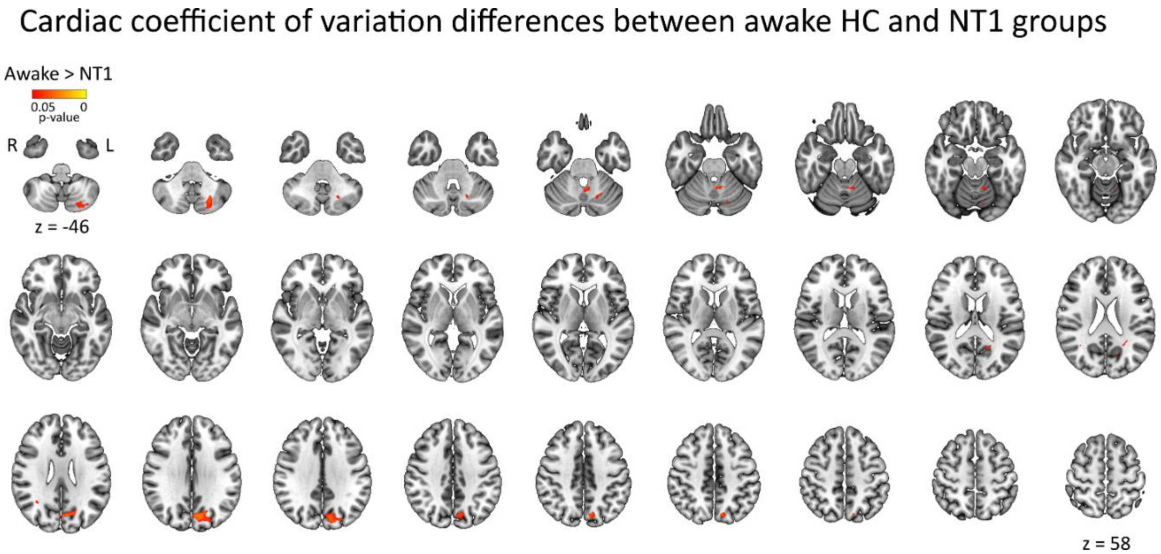

Fig. S6. Spatial distribution of the cardiac coefficient of variation difference maps between the awake HC and NT1 groups (awake HC > NT1). R = right, L = left, z = axial plane MNI152 coordinate.

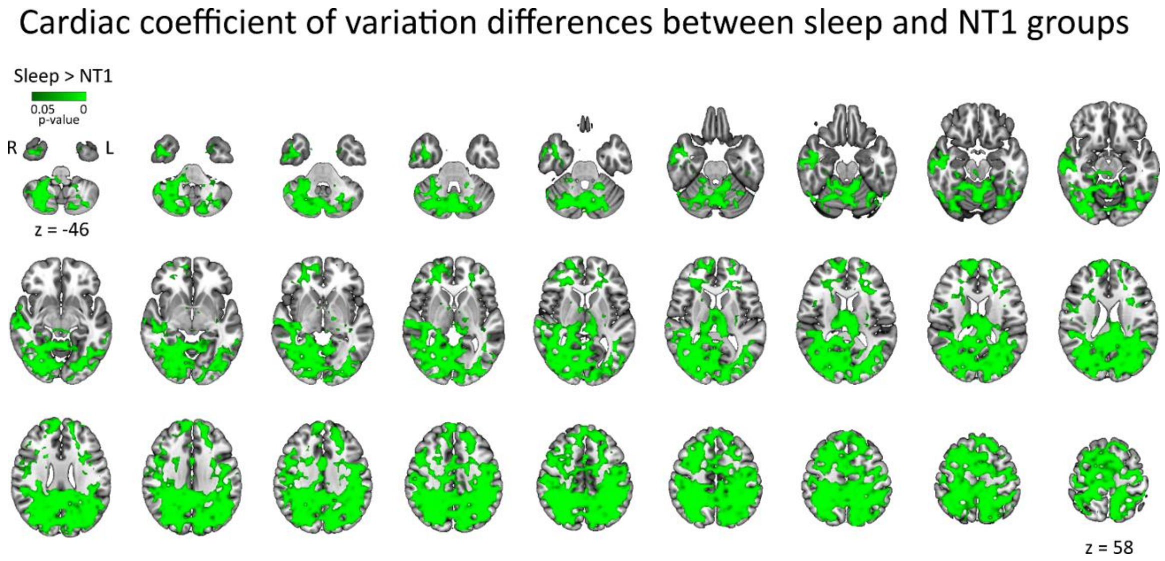

Fig. S7. Spatial distribution of the cardiac coefficient of variation difference maps between the NREM sleep and NT1 groups (NREM sleep > NT1). R = right, L = left, z = axial plane MNI152 coordinate.

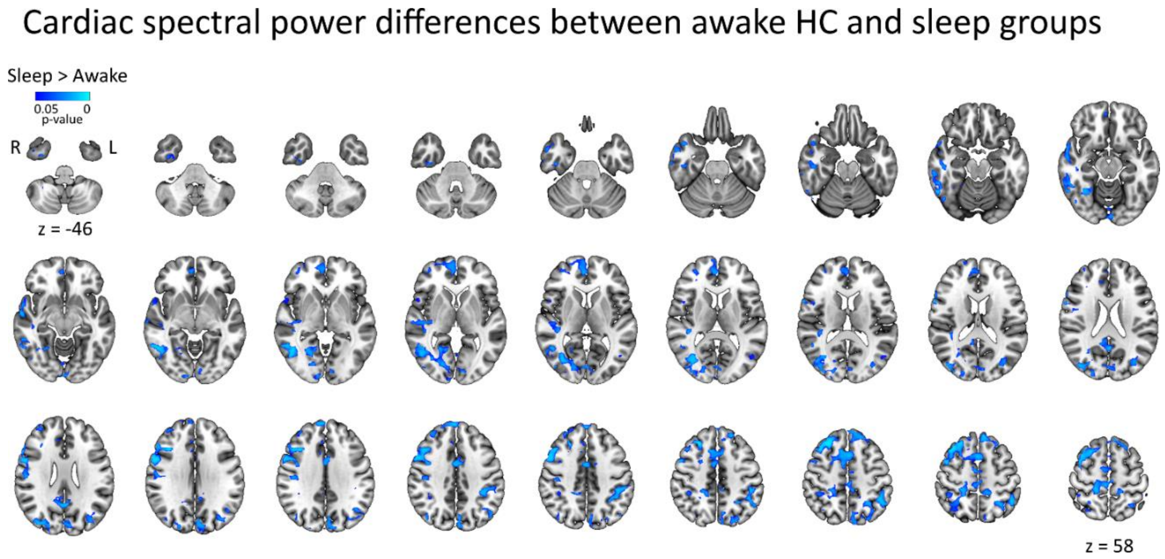

Fig. S8. Spatial distribution of the cardiac spectral power difference maps between the awake HC and NREM sleep groups (NREM sleep > awake HC). R = right, L = left, z = axial plane MNI152 coordinate.

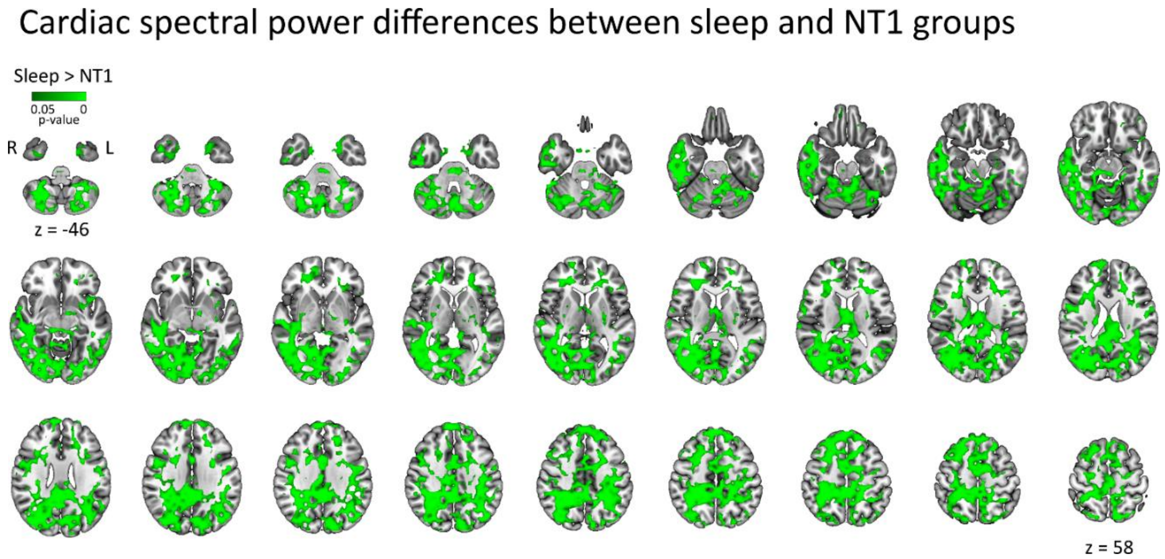

Fig. S9. Spatial distribution of the cardiac spectral power difference maps between the NREM sleep and NT1 groups (NREM sleep > NT1). R = right, L = left, z = axial plane MNI152 coordinate.

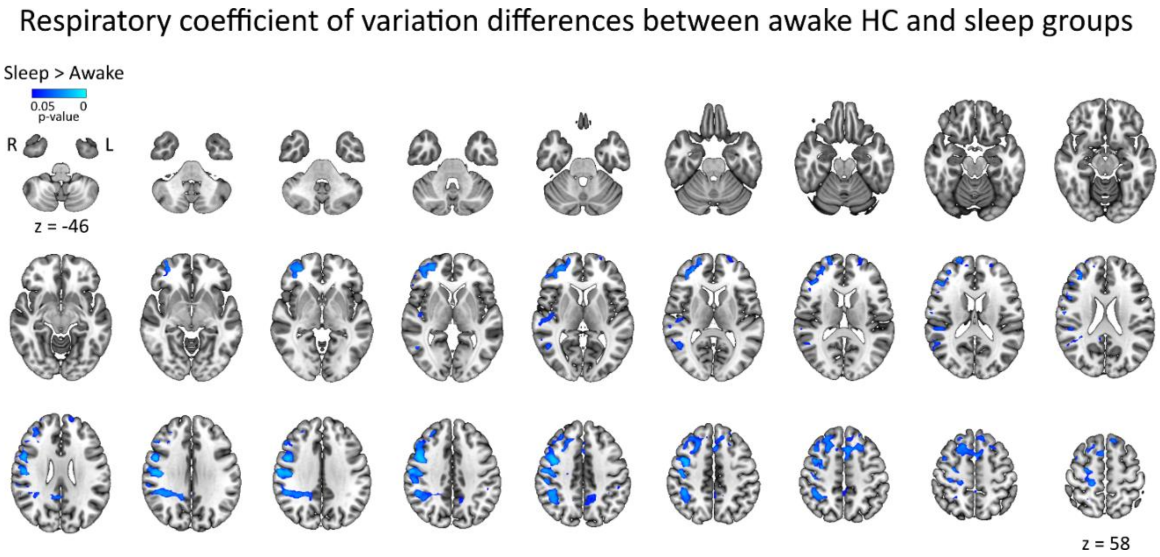

Fig. S10. Spatial distribution of the respiratory coefficient of variation difference maps between the awake HC and NREM sleep groups (NREM sleep > awake HC). R = right, L = left, z = axial plane MNI152 coordinate.

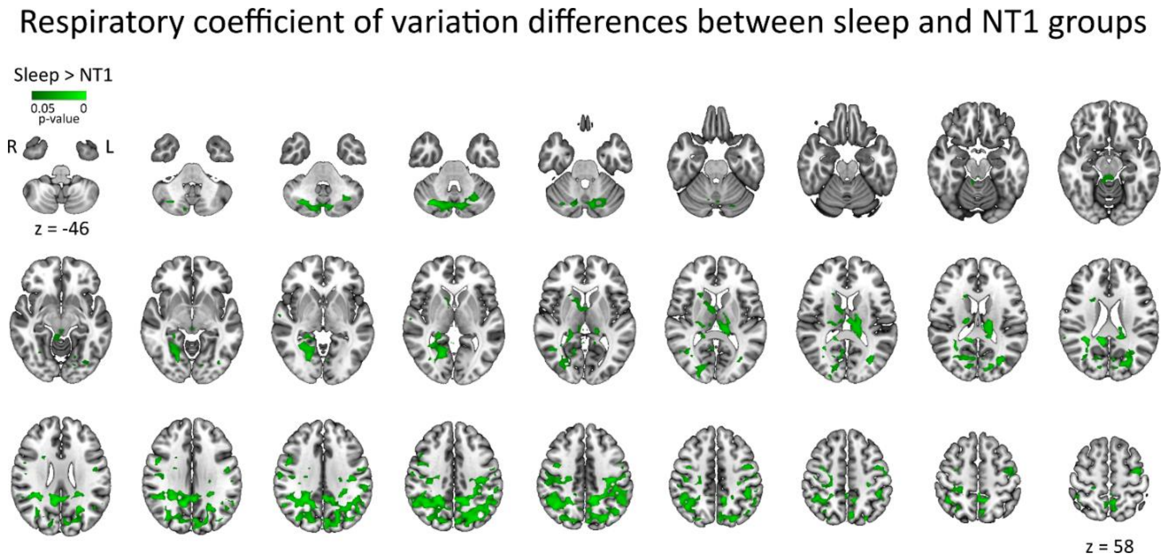

Fig. S11. Spatial distribution of the respiratory coefficient of variation difference maps between the NREM sleep and NT1 groups (NREM sleep > NT1). R = right, L = left, z = axial plane MNI152 coordinate.

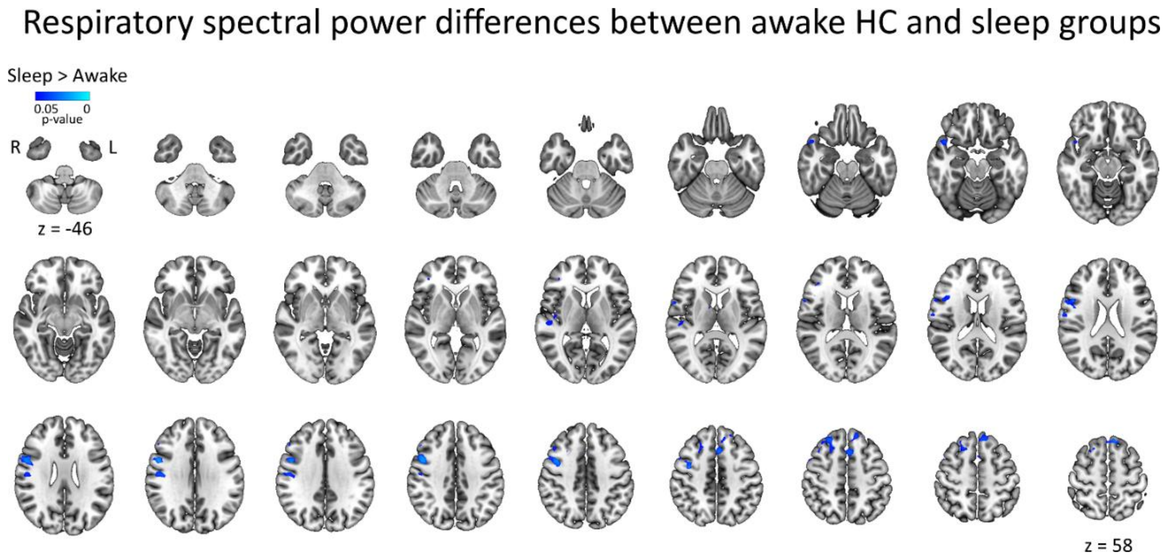

Fig. S12. Spatial distribution of the respiratory spectral power difference maps between the awake HC and NREM sleep groups (NREM sleep > awake HC). R = right, L = left, z = axial plane MNI152 coordinate.

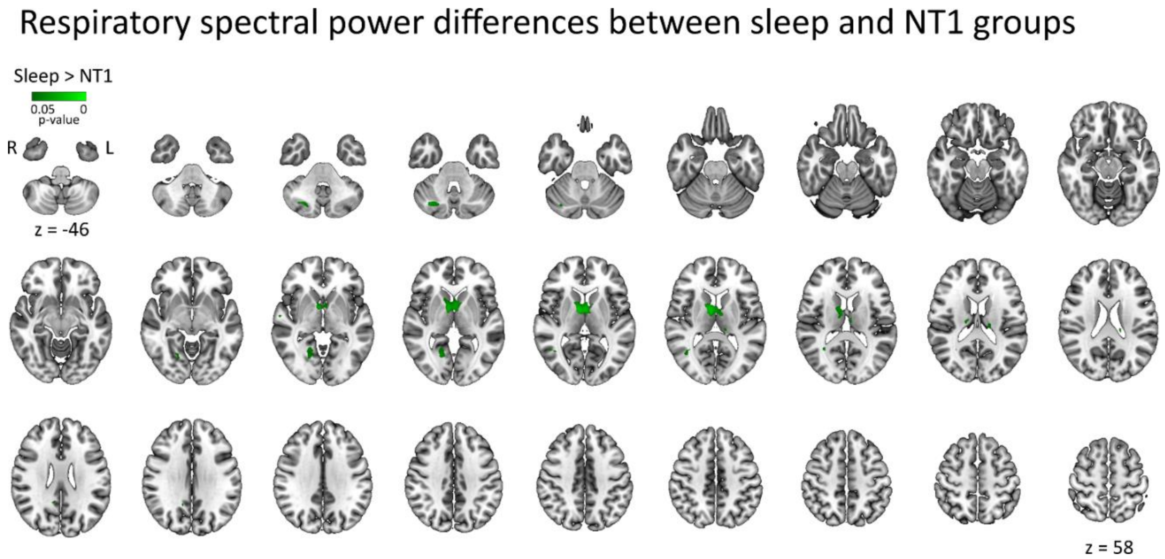

Fig. S13. Spatial distribution of the respiratory spectral power difference maps between the NREM sleep and NT1 groups (NREM sleep > NT1). R = right, L = left, z = axial plane MNI152 coordinate.

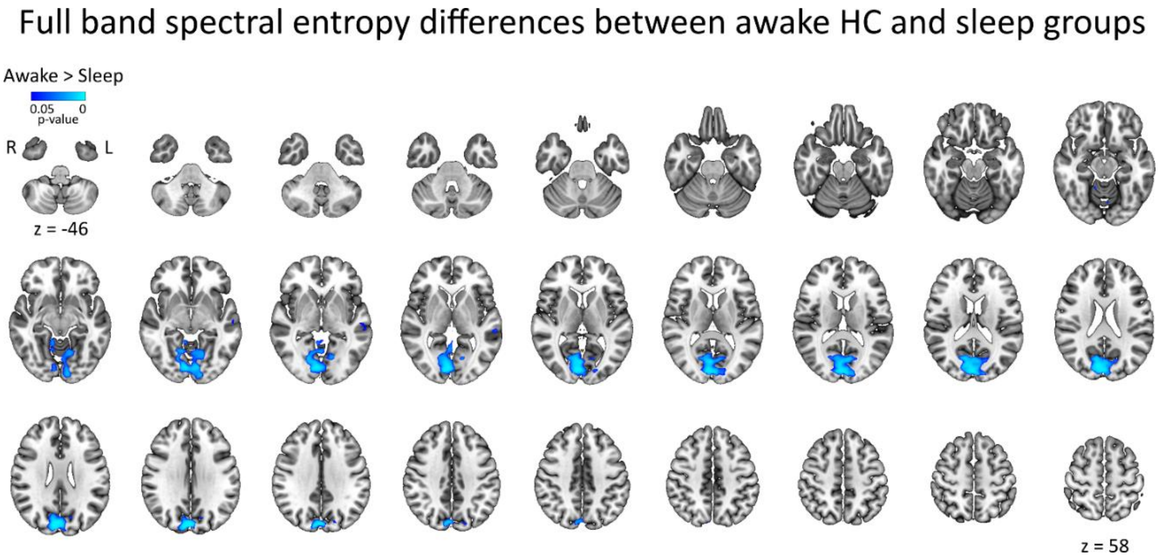

Fig. S14. Spatial distribution of the full band (0.008 – 5 Hz) spectral entropy difference maps between the awake HC and NREM sleep groups (awake HC > NREM sleep). R = right, L = left, z = axial plane MNI152 coordinate.

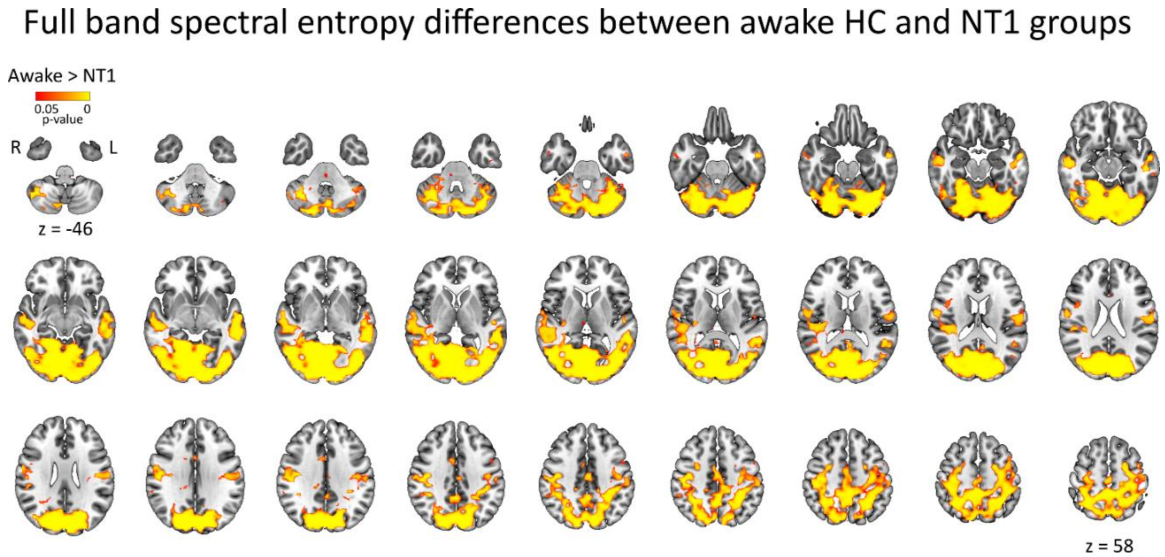

Fig. S15. Spatial distribution of the full band (0.008 – 5 Hz) spectral entropy difference maps between the awake HC and NT1 groups (awake HC > NT1). R = right, L = left, z = axial plane MNI152 coordinate.

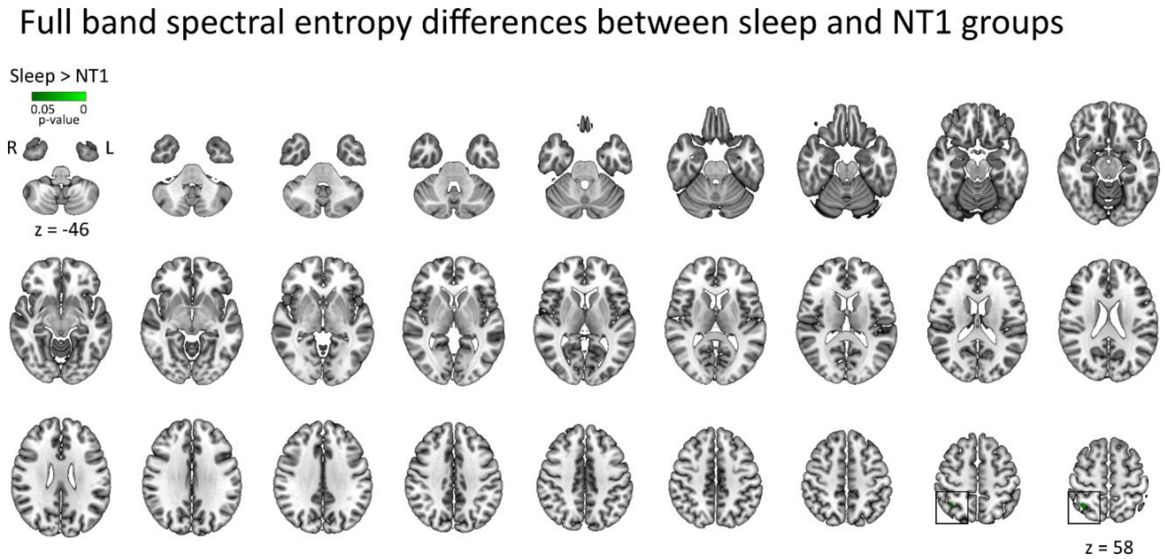

Fig. S16. Spatial distribution of the full band (0.008 – 5 Hz) spectral entropy difference maps between the NREM sleep and NT1 groups (NREM sleep > NT1). R = right, L = left, z = axial plane MNI152 coordinate. The black boxes highlight the area of statistically significant results.

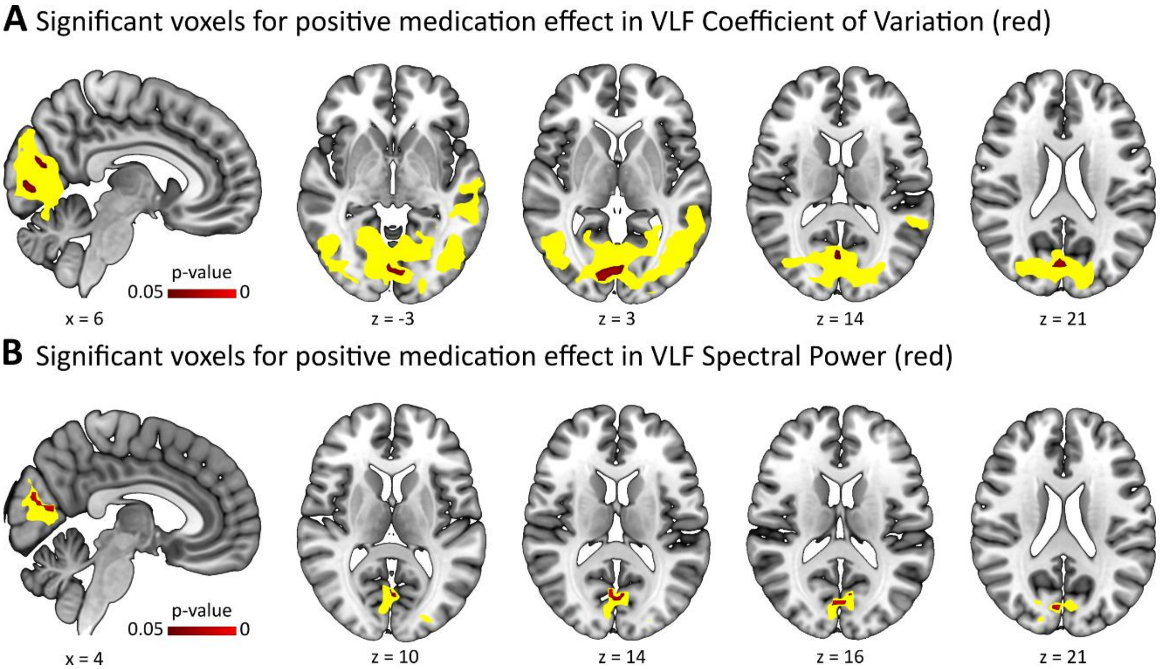

Fig. S17. Voxels where medication status significantly increased VLF Coefficient of Variation (A) and Spectral Power (B) in the weighted model of medication effect on brain pulsations in the NT1 group. The original result (Fig. S2 and S4) is presented as the binarized yellow area to visualize the overlap. $p < 0.05$ = significant, VLF = Very Low Frequencies, x = sagittal and z = axial MNI152 standard space coordinate.

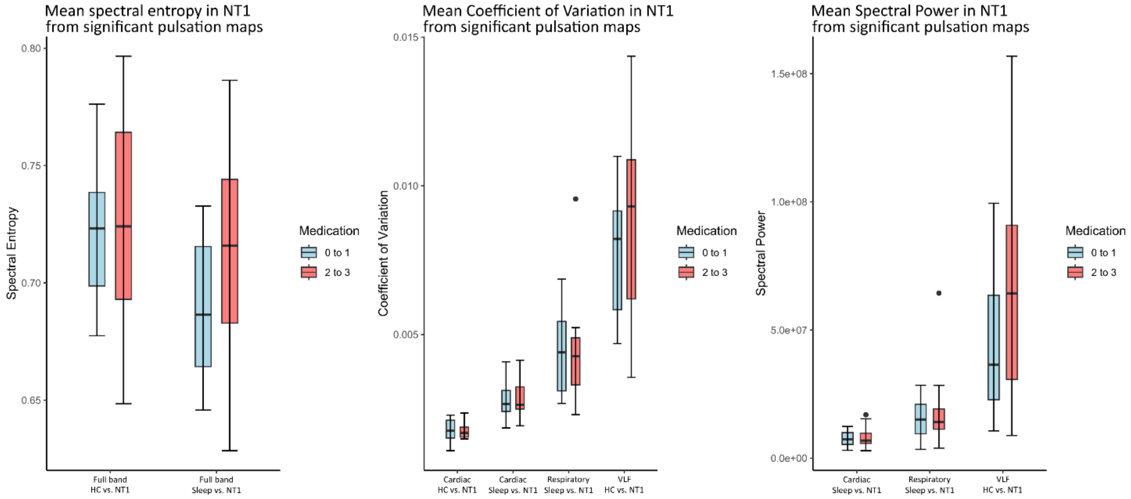

Fig. S18. Box plots of mean pulsation biometric (CV/SP/SE) from NT1 patients as per medication level from significant brain maps including NT1 patients in the main article (nine in total). We fail to find significant differences between the groups.

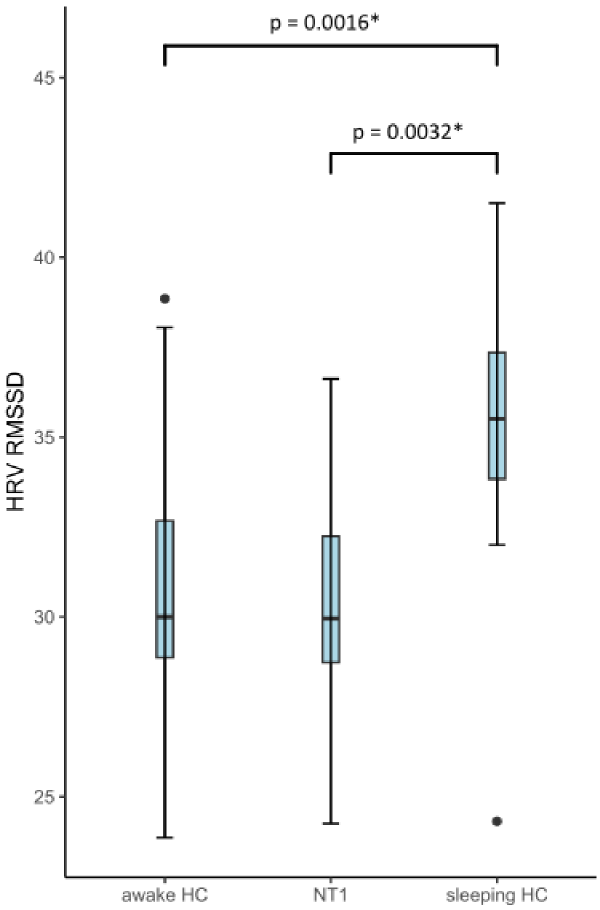

Fig. S19. HRV RMSSD in the study groups. No significant differences were observed between the awake HC and NT1 groups, but sleeping HC had higher RMSSD compared to both other groups. * = significant p-value, $p < 0.05$ = significant.

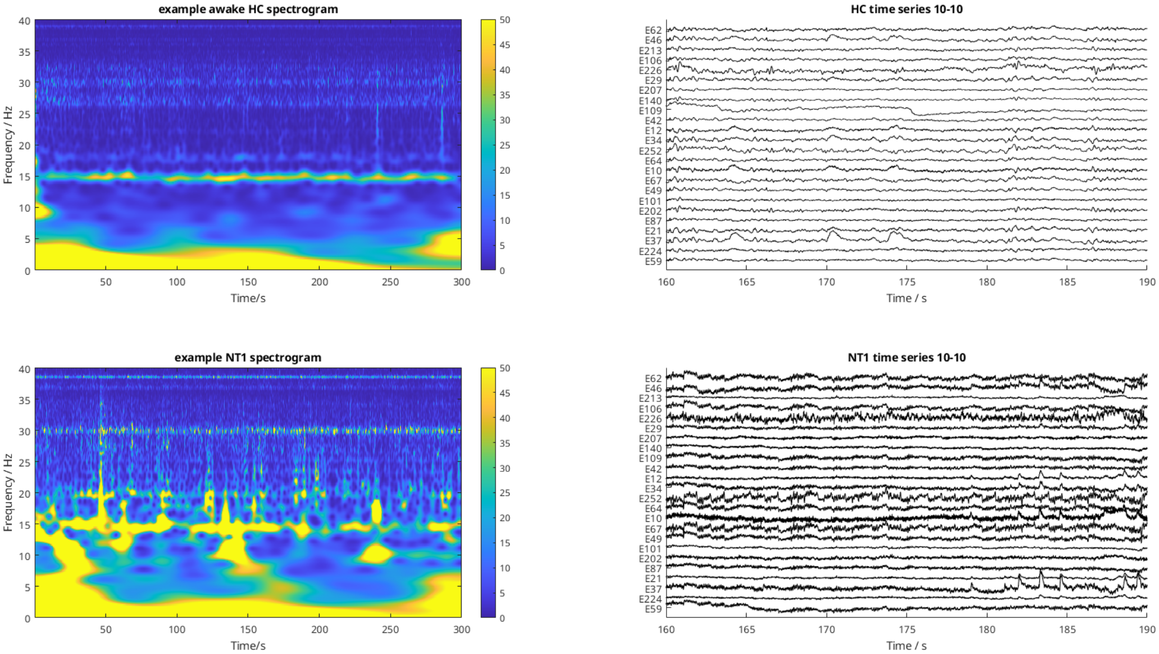

Fig. S20. Example spectrograms and EEG-signals (24 example channels around the scalp using the international 10-10 system) from one awake HC (upper row) and NT1 patient (lower row). By visual observation of the spectrograms, both the alpha band (8 - 12 Hz) and beta band (12.5 - 30 Hz) power fluctuate more (i.e. more timepoints with higher power followed by low power) in the example NT1 subject compared to the awake HC. However statistically, no differences were found between the NT1 group and awake HC group. In the spectrograms, some movement is seen in both subjects as sharp vertical lines (around 50 Hz in NT1 and around 250 and 290 Hz in the awake HC). In the time signals, E marks the channel number. The unit for the spectrogram color bars is mV^2.

Table S1. Effect of flow to MREG derived biometrics

	Baseline	7 cm/s	14 cm/s	21 cm/s
SP	3.4E8 [1.3E8 - 1.2E9]	4.7E9 [1.2E9 - 2.7E10]	8.5E9 [1.5E09 - 3.7E10	9.5E09 [2.3E09 - 4.1E10]
CV	0.016 [0.010 - 0.022]	0.063 [0.030 - 0.11]	0.074 [0.039 - 0.12]	0.076 [0.046 - 0.13]
SE	0.97 [0.90 - 0.99]	0.84 [0.79 - 0.88]	0.84 [0.80 - 0.89]	0.87 [0.82 - 0.93]
SP_b	3.4E8 [1.3E8 - 1.2E9]	1.4E9 [4.3E8 - 5.4E9]	2.7E9 [6.3E8 - 9.9E9]	4.3E9 [1.0E9 - 1.3E10]

Median and interquartile range (in brackets) of SP/CV/SE/SP_b with different mean flow speeds. Baseline SP and SP_b are the same as there is no flow in the system. CV = coefficient of variation, SE = spectral entropy, SP = spectral power, SP_b = background SP, cm = centimeter, s = second.

Table S2. Descriptive statistics

	1)ANOVA or 2) KW p-value	NREM sleep vs. NT1 p-value	awake HC vs. NT1 p-value	awake HC vs. NREM sleep p-value	NT1 group estimates	NREM sleep estimates	Awake HC estimates
Mean region of interest CV	2) 6.1E-09*	3.4E-08*	8.8E-07*	9.7E-03*	1.3E-3 [1.0E-3-1.5E-3]	2.4E-3 [2.2E-3-5.1E-3]	2.0E-3 [1.7E-3-2.8E-3]
Mean respiratory frequency (Hz)	1) 0.29	0.86	0.28	0.80	0.28 ± 0.049	0.27 ± 0.041	0.26 ± 0.060
Mean cardiac Frequency (Hz)	2) 0.010*	0.040*	0.72	0.0074*	1.1 [0.99-1.1]	0.89 [0.84-1.0]	1.1 [0.98-1.1]
Systolic blood pressure (mmHg)	2) 0.11	0.74	0.17	0.45	124 [118-141]	124 [120-139]	133 [126-147]
Diastolic blood pressure (mmHg)	1) 0.48	0.86	0.80	0.49	79 ± 9.5	78 ± 12	81 ± 9.3
Mean arterial pressure (mmHg)	2) 0.27	0.86	0.64	0.64	95 [86-102]	91 [88-103]	98 [91-107]
Mean absolute movement (mm)	2) 0.23	1.0	0.28	1.0	0.11 [0.10-0.14]	0.13[0.10-0.16]	0.14 [0.10-0.20]
Mean relative movement (mm)	2) 0.15	0.17	0.34	0.34	0.025 [0.022-0.027]	0.029 [0.026-0.030]	0.026 [0.023-0.032]
HRV RMSSD	1) 0.0013*	0.0032*	0.92	0.0016*	30.3 ± 3.31	35.3 ± 4.93	30.7 ± 3.18

Global and multiple comparisons corrected post hoc p-values with group-wise descriptive statistics for mean region of interest CV, cardiorespiratory, blood pressure, movement data and HRV RMSSD. CV = coefficient of variation, Hz = hertz, mmHg = millimeters of mercury, mm = millimeter, KW = Kruskal-Wallis test, ANOVA = one-way analysis of variance, HRV RMSSD = heart rate variability root mean square of the successive normal-to-normal intervals, * = significant, $p < 0.05$ = significant. With ANOVA (data normally distributed) mean ± standard deviation and with Kruskal-Wallis (data nonnormally distributed) median and interquartile range are presented.

Table S3. Statistics for blood pressure, cardiac frequency and movement in nonmedicated and medicated NT1 patients

	Systolic (mmHg)	Diastolic (mmHg)	MAP (mmHg)	Mean cardiac frequency (Hz)	Mean absolute movement (mm)	Mean relative movement (mm)
1)T-test, 2)WRS p-value	1) 0.86	1) 0.83	1) 0.99	1) 0.82	2) 0.96	2) 0.31
Nonmedicated NT1	130 ± 21	78 ± 8.1	96 ± 11	1.05 ± 0.059	0.14 [0.12-0.26]	0.020 [0.020-0.032]
Medicated NT1	128 ± 15	80 ± 9.9	96 ± 11	10.6 ± 0.13	0.17 [0.13-0.23]	0.028 [0.026-0.031]

Comparison-wise p-values and test statistics for MAP, systolic, diastolic pressures, mean cardiac frequencies, mean absolute and relative movements. WRS = Wilcoxon Rank-Sum Test, MAP = mean arterial pressure, NT1 = narcolepsy type 1, mmHg = millimeter of mercury, mm = millimeter, $p < 0.05$ = significant. With T-test mean ± standard deviation and with Wilcoxon Rank-Sum Test (data nonnormally distributed) median and interquartile range are presented. No significant p-values were found.

Table S4. Medication information on narcolepsy type 1 patients

Patient	Medication
1	methylphenidate, sodium oxybate
2	modafinil
3	-
4	methylphenidate
5	modafinil, SNRI, sodium oxybate
6	modafinil
7	modafinil, SNRI
8	methylphenidate, sodium oxybate
9	methylphenidate
10	modafinil, methylphenidate
11	modafinil
12	-
13	methylphenidate, sodium oxybate
14	sodium oxibate, SNRI
15	methylphenidate
16	sodium oxibate
17	modafinil
18	modafinil
19	-
20	modafinil, methylphenidate
21	modafinil, SSRI

The medication regimes of NT1 patients. SNRI = serotonin-norepinephrine reuptake inhibitor, SSRI = selective serotonin reuptake inhibitor.

Table S5. Imaging parameters

	MREG	T1
Repetition time (TR)	100 ms	1900 ms
Echo time (TE)	36 ms	2.49 ms
Flip angle (FA)	25°	9°
Inversion time (TI)	-	900 ms
Field of view (FOV)	192	240
Voxel size	(3 x 3 x 3) mm^3	(0.9 x 0.9375 x 0.9375) mm^3

MREG and T1 anatomical imaging parameters.

Table S6. Statistical testing for data normality

	Mean ROI CV SW p-value	Mean respiratory frequency (Hz) SW p-value	Mean cardiac Frequency (Hz) SW p-value	Systolic blood pressure (mmHg) SW p-value	Diastolic blood pressure (mmHg) SW p-value	Mean arterial pressure (mmHg) SW p-value	Mean absolute movement (mm) SW p-value	Mean relative movement (mm) SW p-value
NT1	0.069	0.87	0.34	0.66	0.21	0.76	0.00040*	0.00077*
NREM sleep	0.0023*	0.58	0.1532	0.32	0.47	0.36	0.11	0.00049*
awake HC	0.00081*	0.20	0.035*	0.0013*	0.30	0.020*	9.8e-07*	5.5e-06*

Shapiro-Wilk (SW) test for data normality where significant p-value supports the notion that the data is nonnormally distributed. * = significant p-value, $p < 0.05$ = significant, ROI = region of interest, CV = coefficient of variation, Hz = hertz, mmHg = millimeters of mercury, mm = millimeter.

Table S7. Statistics for mean brain pulsation biometrics between the nonmedicated and medicated NT1 patients

Significant brain maps where mean pulsations were calculated	Fb SE of awake HC vs NT1	Fb SE of NREM sleep vs. NT1	VLF CV of awake HC vs. NT1	Resp CV of NREM sleep vs. NT1	Card CV of awake HC vs. NT1	Card CV of NREM sleep vs. NT1	VLF SP of awake HC vs. NT1	Resp SP of NREM sleep vs. NT1	Card SP of NREM sleep vs. NT1
1)T-test, 2)WRS p-value	1) 0.74	1) 0.14	1) 0.55	2) 0.92	1) 0.97	1) 0.89	1) 0.23	2) 0.99	1) 0.63
0-1 medi-cations	0.72 ± 0.031	0.69 ± 0.032	0.0080 ± 0.0022	0.0044 [0.0031-0.0054]	0.0018 ± 0.00040	0.0028 ± 0.00072	4.6E7 ± 2.9E7	1.5E7 [9.5E6-2.1E7]	7.6E6 ± 3.4E6
2-3 medi-cations	0.73 ± 0.047	0.72 ± 0.048	0.0087 ± 0.0034	0.0043 [0.0033-0.0049]	0.0018 ± 0.00027	0.0029 ± 0.00060	6.7E7 ± 4.8E7	1.4E7 [1.1E7-1.4E7]	8.4E6 ± 4.2E6

Comparison-wise p-values (where 1) = T-test and 2) = Wilcoxon Rank-Sum Test) and descriptive statistics for nonmedicated and medicated NT1 patients. WRS = Wilcoxon Rank-Sum Test, Fb = full band, VLF = very low frequency, Resp = respiratory, Card = cardiac, SE = spectral entropy, CV = coefficient of variation, SP = spectral power, NT1 = narcolepsy type 1, $p < 0.05$ = significant. With T-test mean ± standard deviation and with Wilcoxon Rank-Sum Test (data nonnormally distributed) median and interquartile range are presented. No significant p-values were found.

References

1. J. H. Duyn, Steady state effects in fast gradient echo magnetic resonance imaging. Magnetic Resonance in Med 37, 559–568 (1997).

2. G. K. Von Schulthess, C. B. Higgins, Blood flow imaging with MR: spin-phase phenomena. Radiology 157, 687–695 (1985).

3. N. Huotari, et al., Sampling Rate Effects on Resting State fMRI Metrics. Front. Neurosci. 13, 279 (2019).

4. S. Smith, T. Nichols, Threshold-free cluster enhancement: Addressing problems of smoothing, threshold dependence and localisation in cluster inference. NeuroImage 44, 83–98 (2009).

5. H. Helakari, et al., Human NREM Sleep Promotes Brain-Wide Vasomotor and Respiratory Pulsations. J. Neurosci. JN-RM-0934-21 (2022). https://doi.org/10.1523/JNEUROSCI.0934-21.2022.

6. Mayo Clinic College of Medicine, Rochester, Minnesota, et al., Electroencephalography (EEG): An Introductory Text and Atlas of Normal and Abnormal Findings in Adults, Children, and Infants (American Epilepsy Society, 2016).
